# Addition of MnO_2_ in synthesis of nano-rod erdite promoted tetracycline adsorption

**DOI:** 10.1038/s41598-019-53420-x

**Published:** 2019-11-15

**Authors:** Suiyi Zhu, Yanwen Liu, Yang Huo, Yu Chen, Zhan Qu, Yang Yu, Zhihua Wang, Wei Fan, Juwei Peng, Zhaofeng Wang

**Affiliations:** 1 School of Environment, Northeast Normal University, Changchun, 130117 China; 2 Jilin Institute of Forestry Survey and Design, Changchun, 130022 China; 3 Guangdong Shouhui Lantian Engineering and Technology Co., Ltd, Guangzhou, 510075 China; 4 School of Civil and Environment, Jilin Jianzu University, Changchun, 130117 China; 5 Office of Sponge City Construction and Management, Qingyang, 745099 China

**Keywords:** Environmental sciences, Environmental social sciences

## Abstract

Erdite is a rare sulphide mineral found in mafic and alkaline rocks. Only weakly crystallised fibrous erdite has been artificially synthesised via evaporation or the hydrothermal method, and the process generally requires 1–3 days and large amounts of energy to complete. In this study, well-crystallised erdite nanorods were produced within 3 h by using MnO_2_ as an auxiliary reagent in a one-step hydrothermal method. Results showed that erdite could synthesised in nanorod form with a diameter of approximately 200 nm and lengths of 0.5–3 μm by adding MnO_2_; moreover, the crystals grew with increasing MnO_2_ addition. Without MnO_2_, erdite particles were generated in irregular form. The capacity of the erdite nanorods for tetracycline (TC) adsorption was 2613.3 mg/g, which is higher than those of irregular erdite and other reported adsorbents. The major adsorption mechanism of the crystals involves a coordinating reaction between the −NH_2_ group of TC and the hydroxyl group of Fe oxyhydroxide produced from erdite hydrolysis. To the best of our knowledge, this study is the first to synthesise erdite nanorods and use them in TC adsorption. Erdite nanorods may be developed as a new material in the treatment of TC-containing wastewater.

## Introduction

Erdite is naturally associated with sulphide minerals in mafic and alkali host rocks^1^. Erdite is stable in alkaline conditions and can be observed in the residual sludge obtained from alkaline desulfurisation using sodium ferrite as desulfuriser during the recycling of lead from electrolyte battery waste^2^ and from aluminate salt purification during bauxite refining^3^. Erdite was first synthesised by melting a stoichiometric ratio of Na, S and Fe salts, but the product was intertwined in the form of weakly crystallised fibres^4^. Similar erdite fibres were produced by heating siderite and NaHS at 150 °C, but the hydrothermal process required 2–3 days to complete^5^. Crystallised erdite can be used as raw material in the water, chemical and semiconductor industries^6,7^; therefore, developing new, cost-effective and efficient methods to mass produce the material is highly desirable.

Under acidic and/or neutral pH conditions, erdite is metastable and rapidly decomposes to produce H_2_S and sulphur via the protonation of S-bridges and subsequent redox reaction of Fe(III) –SH groups^8^. Lassin *et al*.^2^ investigated the dissolution–precipitation of lead-refining slag (containing 20–50% erdite) from waste battery disposal and found that the final solution is alkaline; in addition, the predominant species were Na^+^, Fe(OH)_4_^−^, HS^−^ and SO_4_^2−^. The breakdown of erdite may generate fresh Fe oxyhydroxide, which has a large number of surface coordination sites and could be used to adsorb pollutants in wastewater^9^. However, the removal of pollutants in wastewater by using erdite has yet to be studied.

Pharmaceutical tetracycline (TC) is used worldwide in food additives, human therapy and farming industry^10^. Because TC is poorly metabolised, large fractions of the drug are excreted through urine and faeces as the unmodified parent compound^11^. In conventional biological wastewater treatment, TC is mainly removed by biosorption, but only small portions of it are biodegraded^12,13^. The discharge limit and acceptable level of TC have not been published^14^. To avoid TC pollution, Chinese government strengthened regulations to limit the chemical oxygen demand of the treated effluent to less than 50 mg/L^14,15^. TC forms residues and is frequently detected in soil and surface waters near the discharge outlet of pharmaceutical wastewater treatment plants^16,17^. Many approaches, including wet oxidation, electrochemical treatment, photocatalysis^18^, adsorption^19,20^, coagulation and chlorination, have been developed to effectively remove TC from wastewater. Advanced oxidation employs free radicals to nonselectively oxidise organic matter (e.g. TC)^21,22^ without generating secondary pollutants^23^; its efficiency can be improved by enhancing the photocatalyst activity and/or reaction conditions^24–26^, but its applications are limited by high energy consumption and complicated instrumentation^27^. As an alternative to this method, adsorption is characterised by high effectiveness, cost efficiency and simply operation^28^; thus, it can be applied to treat TC-bearing wastewater. Various natural minerals and synthesised materials, such as montmorillonite^29^, activated carbons^30^, active sludge^12^, graphene^31^ and Fe–Mn binary oxide^32^, have been used to remove TC from wastewater. The adsorption performance of sorbents can be considerably improved by increasing the availability of surface sites and/or activating surface functional groups. Thus, the development of efficient adsorbents for TC removal remains an urgent undertaking.

In this study, we synthesised well-crystallised erdite nanorods via a one-step hydrothermal method using MnO_2_ as an auxiliary reactant. Using the proposed method, erdite nanorods were generated within only 3 h. The synthesised erdite nanorods showed high capacity for TC adsorption and, therefore, great application potential in wastewater treatment.

## Results and Discussion

**Figure 1:**
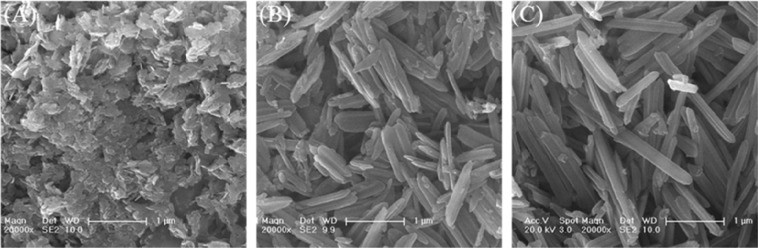
SEM images of erdite particles synthesised at Mn/Fe molar ratios of (**A**) 0, (**B**) 0.05 and (**C**) 0.2.

**Figure 2:**
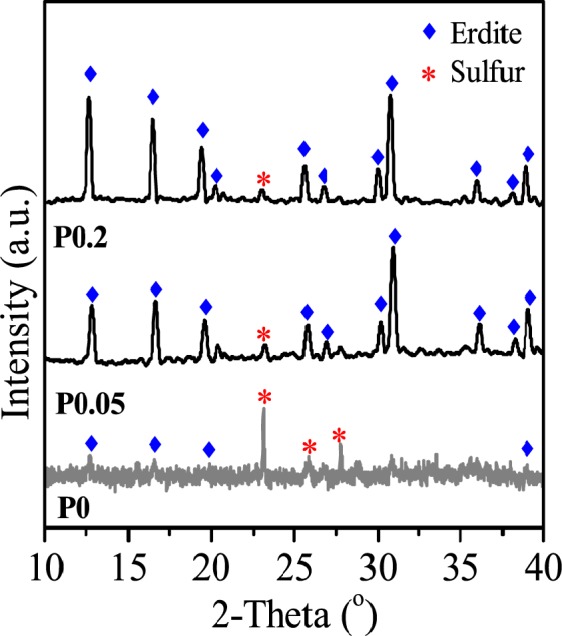
XRD spectra of erdite particles synthesised at Mn/Fe molar ratios of (P0) 0, (P0.05) 0.05 and (P0.2) 0.2.

The morphology and crystallography of the erdite particles were recorded by Scanning electron microscope (SEM) and X-ray powder diffraction (XRD) . Figure 1(A) shows that, without MnO_2_, the prepared P0 presents as irregular flake-shaped flocs. After MnO_2_ addition, nanorod particles with a diameter of approximately 200 nm and lengths of 0.5–2 μm are formed (Fig. 1(B)). The weak diffraction peaks of P0 (Fig. 2 P0) at 2θ = 12.7°, 16.5°, 19.4° and 39°, which correspond to the (110), (200), (020) and (330) crystal planes of erdite (JCPDS 83–1323), respectively, intensified in P0.05 and P0.2 (Fig. 2, P0.05 and P0.2), thus suggesting that erdite nanorods are crystallised by MnO_2_ addition and that the crystals grow with increasing addition of MnO_2_.

When the Mn/Fe molar ratio was increased from 0.05 to 0.2, crystallisation was improved, and the length of the nanorod crystals increased to 3 μm (Fig. 1(C)). Moreover, the predominant orientation of the erdite phase of P0.2 along the (110) reflection intensified (Fig. 2) in comparison with that of P0.05. This finding suggests that excess MnO_2_ promotes microcrystal growth along the (110) reflection to produce the long erdite nanorods of P0.2. A peak at 2θ = 23.1°, which belongs to S_8_ (JCPDS 78-1888), was observed in all three spectra, thereby indicating that the polysulphide S_8_ is generated in the formation of erdite.

**Figure 3:**
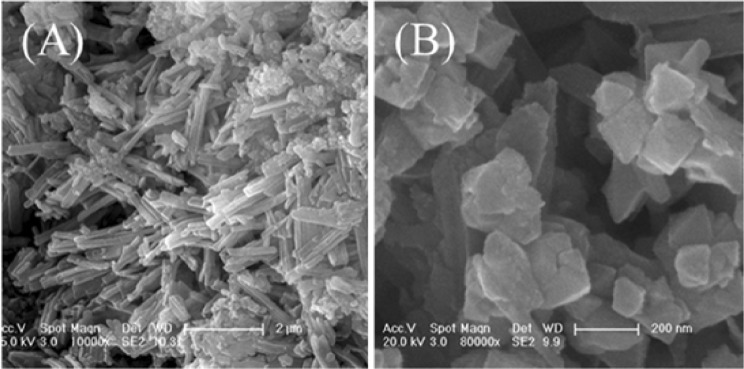
SEM images of erdite particles synthesised at a Mn/Fe molar ratio of 1.

**Figure 4:**
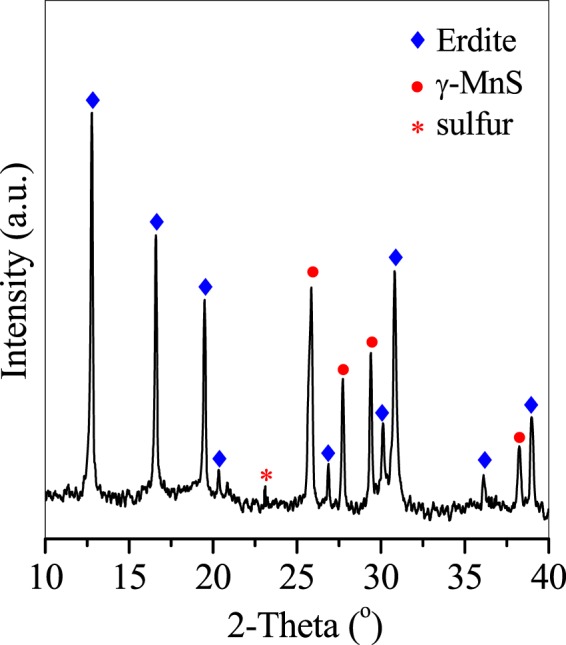
XRD pattern of erdite particles synthesised at a Mn/Fe molar ratio of 1.

When the Mn/Fe ratio was increased to 1, a mixture of nanorods (Fig. 3(A)) and octahedral and irregular particles was obtained (Fig. 3(B)). The nanorod particles showed a morphology similar to that of P0.2 and was attributed to well-crystallised erdite (Fig. 4); the octahedral and irregular particles were considered to belong to γ-MnS and polysulphide S_8_ (Fig. 4), respectively. This result demonstrates that excess MnO_2_ at a molar ratio of 1 maintains the morphology of erdite nanorods but increases the content and size of γ-MnS crystals.

**Figure 5:**
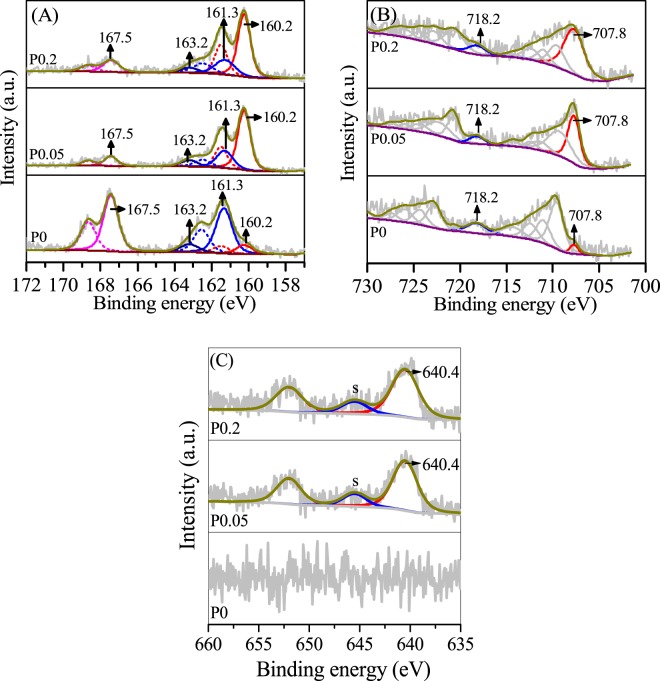
High-resolution (**A**) S 2p, (**B**) Fe 2p and (**C**) Mn 2p XPS spectra of P0, P0.05 and P0.2.

The valences of S, Fe and Mn on the surface of the erdite particles were determined by X-ray photoelectron spectrometry (XPS). The S 2p spectra in Fig. 5(A) reveal four major S 2p3/2 peaks at 160.2, 161.3, 163.2 and 167.5 eV; these peaks belong to structural S_2_ in erdite, S^2−^, polysulphide and SO_4_^2−^, respectively. Deconvolution of the S 2p envelope indicates that the relative area of the S^2−^ peaks is 26.5% in P0 but only 15.6% in P0.2. By contrast, the relative area of structural S_2_ peaks in erdite increased from 5.2% in P0 to 44.3% in P0.2. This result indicates that S^2−^ is involved in the formation of erdite. The Fe 2p 3/2 spectra of P0 in Fig. 5(B) show a weak peak at 707.8 eV, which could be assigned to Fe^3+^–S in erdite; this peak intensified in P0.05 and P0.2. The results indicate that erdite generation is enhanced by addition of MnO_2_, consistent with the XRD results (Fig. 2). The Mn 2p spectra showed a peak with a binding energy of 640.4 eV and a satellite peak 5.1 eV ahead of the binding energy (Fig. 5(C)), which may be assigned to structural Mn^2+^ in MnS^33,34^. These results reveal that reduction of MnO_2_ by Na_2_S, along with the generation of MnS, occurs in P0.05 and P0.2.

When Na_2_S is added to the liquid phase, it hydrolyses to HS^−^ and OH^−^, resulting in increases in pH. When the pH of the solution is greater than 13, Fe^3+^ is hydrolysed to Fe(OH)_4_^−^ ^35^. HS^−^ is diffused into the structure of Fe(OH)_4_^−^, and OH^−^ is replaced by HS^−^ with the formation of Fe(OH)_3_HS^−^ (Eq. (1)). The two newly formed Fe(OH)_3_HS^−^ species are subsequently polymerised with the release of two water molecules (Eq. (2)). The polymerisation reaction continues in the presence of adequate Fe(OH)_4_^−^, with the polymer chain (FeS_2_)_n_^n−^ being the final product (Eq. (3)). The free charges on the (FeS_2_)_n_^n−^ chain are neutralised by Na^+^ ^36^, and free channels within these chains are occupied by water molecules^37^. Therefore, well-crystalised erdite consisting of long linear (FeS_2_)_n_^n−^ chains, with each Fe ion tetrahedrally surrounded by four sulphur atoms^38^, is generated.

1 $$\text{Fe}{({\rm{O}}{\rm{H}})}_{{\rm{4}}}^{-}+{\rm{H}}{S}^{-}\leftrightarrow {\rm{F}}e{({\rm{O}}{\rm{H}})}_{{\rm{3}}}\,{\rm{H}}{{\rm{S}}}^{-}+{\rm{O}}{{\rm{H}}}^{{\rm{-}}}$$

23

In Eq. (3), R_1_ and R_2_ represent the growing polymeric species of the (FeS_2_)^−^ unit.

During erdite formation, replacement of OH^−^and polymerisation of (FeS_2_)_n_^n−^ (Eqs (1–3) occur spontaneously and rapidly^3^, and the formed (FeS_2_)_n_^n−^ chain is stable in structure, even after calcination at approximately 500 °C for 12 h^39^. This finding indicates that the redox reaction of Fe–S in erdite is considerably inhibited.

Dissolved oxygen in the liquid phase reacts with HS^−^ to form elemental sulphide and OH^−^ as the byproduct (Eq. 4). Elemental sulphur is a key intermediate^10,40^ and could react with HS^−^ to form polysulphide S_8_ via Eqs (5, 6) ^41^. Polysulphide S_8_ has low solubility in solution^10^ and forms in the colloidal state at high pH^17,41^, followed by precipitating from the aqueous solution^42^. Oxidation of HS^−^ to sulphate also occurs under oxygen-rich conditions via Eq. (7)^43^ but is inhibited by the exhaustion of dissolved oxygen during the hydrothermal process. Luther *et al*., using a frontier molecular orbital model, postulated the formation of elemental S as an intermediate in the H_2_S and H_2_O_2_ reactions and found the formation of polysulphide S_8_ as the major end product as well as a small amount of sulphate as a minor product^43,44^.

4 $$2H{S}^{-}+{O}_{2}\to 2S+2O{H}^{-}$$

5 $$H{S}^{-}+S\to H{S}_{2}^{-}$$

6 $$H{S}_{2}^{-}+7S\to {S}_{8}+H{S}^{-}$$

7 $$H{S}^{-}+2{O}_{2}+O{H}^{-}\to S{O}_{4}^{2-}+{H}_{2}O$$

Addition of MnO_2_ accelerates the oxidation of HS^−^ via the following reactions. HS^−^ is rapidly oxidised to elemental sulphide with the generation of MnOOH and OH^−^ via Eq. (8) ^45^. Thus, the reaction of HS^−^ and dissolved oxygen is catalysed by the insoluble MnOOH formed under alkaline conditions^41,45^, which promotes the formation of polysulphide S_8_ via Eqs (5, 6).

8 $${\rm{H}}{S}^{-}+2Mn{O}_{2}+{H}_{2}O\to 2MnOOH+S+O{H}^{-},$$

Therefore, a large amount of OH^−^, which promotes the formation of Fe(OH)_4_^−^ and polymerisation of (FeS_2_)_n_^n−^ chains, is produced as a result of the three reactions (Eqs (1, 4 and 8)). When the dissolved oxygen is exhausted, the structural Mn in MnOOH is readily reduced to Mn^2+^ by HS^−^ ^46^ with the generation of MnS. Without MnO_2_, oxidation of HS^−^ is slow, leading to the low production of OH^−^ and Fe(OH)_4_^−^, which inhibits erdite formation.

The adsorption capacity of the erdite nanorods for TC was investigated because the latter is a common pollutant in pharmaceutical wastewater^19,29^. Four of the most widely established isotherm models, namely, the Langmuir (Eq. (9)), Freundlich (Eq. (10)), Dubinin–Radushkevich (D–R) (Eq. (11)) and Temkin (Eq. (12)) isotherms, were used to simulate the experimental data; these models can be expressed as follows:

9 $$\frac{{C}_{e}}{{q}_{e}}=\frac{1}{{K}_{L}{q}_{m}}+\frac{{C}_{e}}{{q}_{m}},$$

10 $$ln{q}_{e}=ln{K}_{F}-{b}_{F}ln{C}_{e},$$

11 $$ln{q}_{e}=ln{q}_{m}-{K}_{DR}{\varepsilon }^{2},$$

12 $${q}_{e}=BlnA+Bln{C}_{e},$$

where *C*_e_ is the equilibrium concentration of TC in the solution (mg/L), *q*_e_ is the equilibrium adsorption capacity (mg/g), *K*_L_ and *q*_m_ are the Langmuir isotherm constant and maximum adsorption capacity (mg/g), respectively, *K*_F_ and *b*_F_ are the Freundlich constants, *K*_DR_ is the D–R isotherm constant and *A* and *B* are the Temkin isotherm constants.

**Figure 6:**
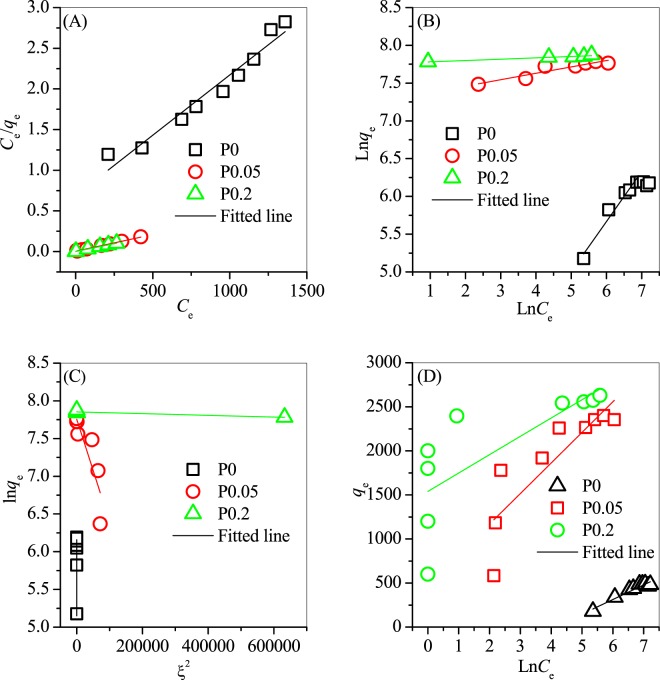
Linear fitting of the (**A**) Langmuir, (**B**) Freundlich, (**C**) D–R and (**D**) Tempkin isotherms to describe the adsorption of TC on the prepared erdite particles.

The linear fittings of the four isotherm models are shown in Fig. 6. The parameters of each isotherm model were calculated with their correlation coefficients (*R*^2^) and the results are shown in Table 1. The fitting of the linearised Langmuir model to the experimental data was better than those of the Freundlich, D–R and Temkin isotherms. Thus, the Langmuir isotherm provides the best description of the experimental data.

**Table 1:** Parameters and regression coefficients (R^2^) of the isotherm models.

Isotherm	Parameters	P0	P0.05	P0.2
Langmuir	R^2^	0.972	0.997	0.991
	*q*_*m*_ (mg/g)	675.7	2446.7	2613.3
	*K*_*L*_	0.004	0.088	0.097
Freundlich	R^2^	0.882	0.867	0.937
	*b*_*F*_	0.521	0.085	0.068
	*K*_*F*_	12.7	1467.6	2352.4
D–R	R^2^	0.951	0.764	0.819
	*q*_*m*_ (mg/g)	484.9	2336.2	2575.3
	*K*_*DR*_	0.008	1.36 × 10^−5^	1.51 × 10^−5^
Templin	R^2^	0.927	0.711	0.630
	B	166.1	346.9	408.1
	A	0.016	3.92	4.31

**Figure 7:**
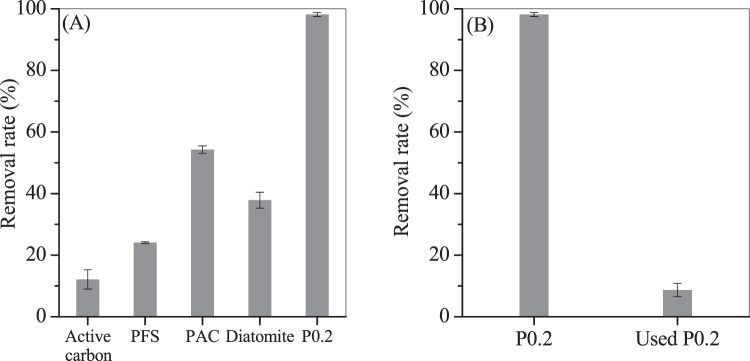
(**A**) Adsorption capacities of TC by P0.2, activated carbon powder, PAC, PFS and diatomite. (**B)** Reusability of P0.2 for TC adsorption. Experimental conditions: TC concentration = 1000 mg/L, adsorbent dosage = 0.01 g, equilibrium time = 2 h.

The maximum adsorption capacity of erdite increased significantly from 675.7 mg/g for P0 to 2446.7 mg/g for P0.05 and then to 2613.3 mg/g for P0.2; these results indicate that MnO_2_ is important to the adsorption capacity of erdite for TC. MnO_2_ is a common auxiliary reagent and widely used to promote the performance of adsorbents toward various contaminant (e.g. TC)^31,47^. For instance, Song *et al*. reported that graphene loaded with 40% nanorod MnO_2_ exhibits a maximum TC adsorption capacity (*q*_m_) of 1789 mg/g and that the observed adsorption capacity remains constant with increasing MnO_2_ loadig from 40% to 60%^31^. In our study, P0.2 showed a desirable TC *q*_m_ of 2613.3 mg/g, which is higher than those of P0, 40% MnO_2_-loaded graphene^31^, carbon materials (maximum of 1340.8 mg/g)^30^ and zeolite materials (800 mg/g)^19^. P0.2 also showed a TC removal rate higher than those of other flocculants (Fig. 7(A)), such as polymeric aluminium chloride (PAC) and polymeric ferric sulphate (PFS). Similar to these flocculants, used P0.2 has an amorphous form and shows a lower TC removal rate in comparison with that of P0.2 (Fig. 7(B)); this finding suggests that the reusability of erbite particles is undesirable. After adsorption, the solution pH slightly increased to approximately 7.4, but the concentrations of Fe, Mn, sulphate and sulphide did not change obviously. Hence, no secondary pollutant was released to the solution during adsorption.

**Figure 8:**
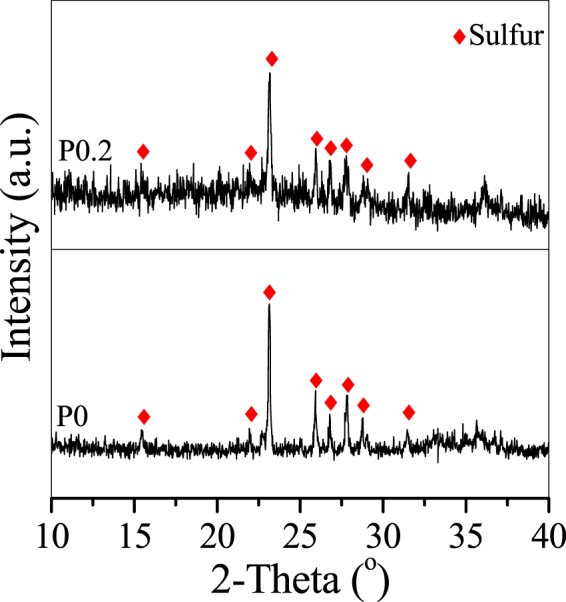
XRD spectra of P0 and P0.2 after TC adsorption.

**Figure 9:**
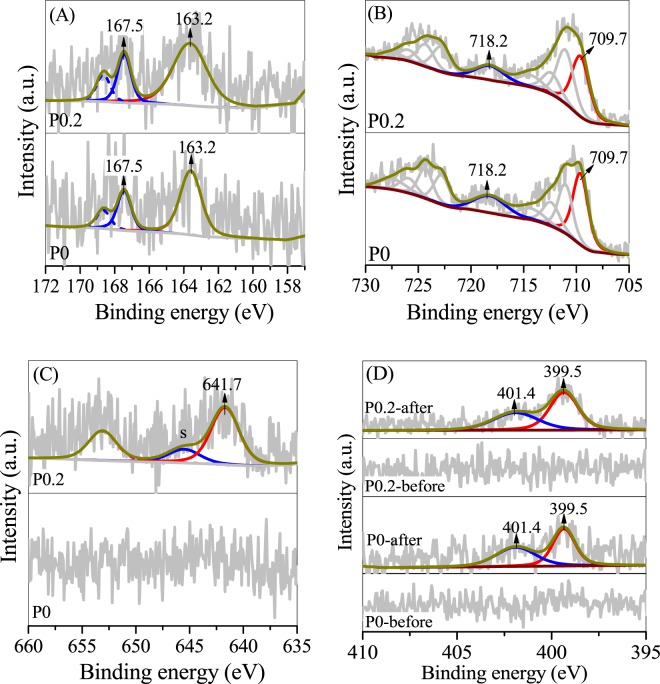
High-resolution (**A**) S2p, (**B**) Fe 2p, (**C**) Mn 2p and (**D**) N 1s XPS spectra of P0 and P0.2 after TC adsorption.

P0 and P0.2 were characterised by XRD and XPS after adsorption to investigate the adsorption mechanism of TC by erdite. The diffraction peaks of erdite intensified from P0 to P0.2, as shown in Fig. 3, but disappeared after TC adsorption, leaving only S_8_ diffraction peaks (Fig. 8). Thus, only the XPS peaks of polysulphide and sulphate are recorded in Fig. 9(A). The Fe 2p XPS spectra (Fig. 9(B)) shows the absence of the typical peak of Fe(III) -S in erdite at 707.8 eV and the formation of a new peak at 709.7 eV, which is attributed to Fe oxyhydroxide from erdite hydrolysis. These findings indicate that the erdite in P0 and P0.2 is completely hydrolysed with the formation of weakly crystallised Fe oxyhydroxide. The Mn 2p3/2 peak with a binding energy of 641.7 eV (Fig. 9(C)) agrees with the Mn^3+^ peak in MnOOH reported in other studies^48–50^, thus suggesting that the hydrolysis of MnS in P0.2 is followed by oxidation during TC adsorption^45^. The N 1 s XPS spectra (Fig. 9(D)) of P0 and P0.2 show two peaks at 399.5 and 401.4 eV, which belong to the N atoms of –NH_3_^+^ and –NH– of TC, respectively, after adsorption. No peak was observed in the N1s spectra of P0 and P0.2 before adsorption, thus suggesting that the adsorption of TC occurs on the hydrolysed species of P0 and P0.2.

**Figure 10:**
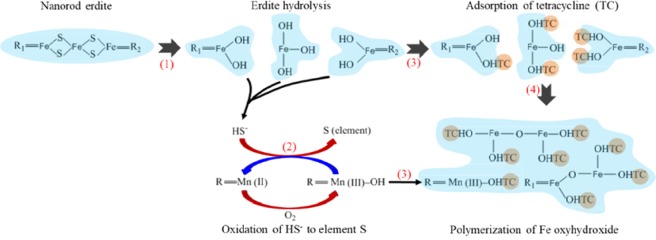
Illustration of TC adsorption by erdite particles.

When erdite is added to TC-containing solution, hydrolysis of erdite probably proceeds by protonation of the S–Fe bond. Subsequently, (FeS_2_)_n_^n−^ chains are disintegrated via the reverse reaction of Eq. (1) (Fig. 10, step 1) to yield Fe oxyhydroxide as the final product because Fe(OH)_4_^−^ is unstable and rapidly dehydroxylated at pH < 11. No S^2−^-containing compounds are observed in the XPS S 2p spectra (Fig. 9(A)), which suggests that the concentration of surface S^2−^ is low. Thus, the redox reaction of the Fe-S bond in erdite is subordinate in comparison with the hydrolysis of erdite. MnS is similarly hydrolysed, and the Mn^2+^ obtained is oxidised to MnOOH in the presence of dissolved oxygen^51^, which oxidises HS^−^ at ambient condition (Fig. 10, step 2)^45^.

Addition of MnO_2_ during erdite synthesis enhanced TC adsorption. Well-crystallised erdite (P0.2) could be generated by addition of MnO_2_, which is subsequently hydrolysed to generate more Fe oxyhydroxide and MnOOH (Fig. 10(B,C)). Both products have abundant surface hydroxyl groups^48,52^, which serve as coordination sites for TC adsorption (Fig. 10, step 3). In the liquid phase, TC forms an amphoteric ion^30^, which is readily adsorbed by Fe oxyhydroxide and MnOOH^29^. The –NH_2_ group in the side chain of TC links to H in the hydroxyl group of Fe oxyhydroxide and MnOOH to form the –NH_3_^+^ group, resulting in TC adsorption. As erdite hydrolysis continues, polymerisation of two adjacent Fe oxyhydroxides occurs with the release of a water molecule, resulting in the formation of irregular aggregates (Fig. 10, step 4). The uniform P0.2 nanorod particles are apparently smaller than the P0 flocs (Fig. 1(A,C)), but the hydrolysis product of erdite in P0.2 (i.e. Fe oxyhydroxide) shows better TC adsorption in comparison with that of the P0 flocs.

Erdite shows unique characteristics in acidic or neutral pH solution and could generate Fe oxyhydroxide, which plays a key role in TC adsorption after its hydrolysis. The product could also adsorb various other pollutants, such as trace heavy metals^53^, dissolved organic matter^54^ and bacteria^55^; such properties confer this novel material with promising potential for application to the adsorption of heavy metals and organics from wastewater. Similar to FeCl_3_, during erdite production, solid waste containing Fe oxides, such as goethite, hematite and magnetite, could be reduced by Na_2_S^56^. These wastes ubiquitously exist in groundwater treatment sludge^57,58^, red mud and fly ash^59^ and could significantly decrease the cost of erdite production. Overall, our results demonstrate an alternative strategy for recycling Fe-containing solid waste for low-cost erdite production. Future studies could investigate the synthesis of erdite from recycled Fe-containing waste and test the effectiveness of the resulting material in wastewater treatment.

## Materials and Methods

### Synthesis of erdite nanorods

**Figure 11:**
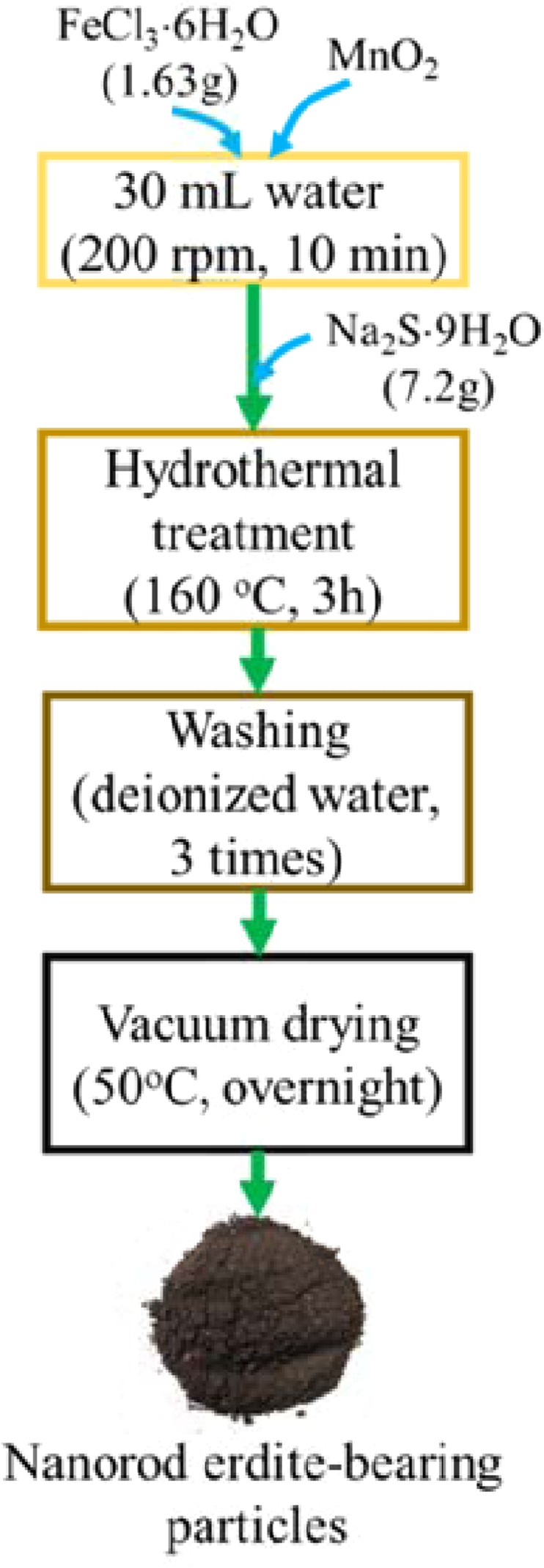
Flow chart of the synthesis of erdite particles.

Erdite nanorods were hydrothermally synthesised via the following steps (Fig. 11). In brief, 1.63 g of FeCl_3_·6H_2_O was dissolved in 30 mL of deionised water and then added with MnO_2_ powder under magnetic stirring. After stirring for 10 min at 200 rpm, the suspension produced was transferred to a 50 mL Teflon vessel and added with 7.2 g of Na_2_S.9H_2_O. The vessel was then sealed and hydrothermally treated at 160 °C for 3 h before cooling down to room temperature. The deposits at the bottom of the vessel were collected, washed five times with 30 mL of deionised water, and then vacuum dried at 50 °C overnight. During erdite synthesis, the Mn/Fe molar ratio was varied from 0 to 0.05 to 0.2, and the obtained erdite particles were denoted as P0, P0.05 and P0.2, respectively.

### TC adsorption by the erdite nanorods

TC, a typical antibiotic found at high levels (above 920 mg/L) in pharmaceutical wastewater^12^, was employed to assess the adsorption performance of the synthesised erdite nanorods. A stock solution of 2000 mg/L TC was prepared, and its pH was adjusted to 5 by adding 1.44 mol/L HCl and 1.32 mol/L NaOH. A series of dilute solutions was prepared from the stock by addition of deionised water. Each dilution (20 mL) was mixed with 0.01 g of the erdite particles in an Erlenmeyer flask. The flasks were sealed with parafilm and shaken at 200 rpm in an incubator (THZ-98A, Yiheng, Shanghai, China) at room temperature. After 24 h, the flasks were taken from the incubator, and the erdite particles were separated by agitation at 5500 rpm for 5 min. The TC concentration in the supernatant was determined by high-performance liquid chromatography (LC-16, Shimadzu, Japan) using a mobile phase composed of 0.1% phosphoric acid and methanol at a ratio of 60:40 (v/v). The absorbance at 268 nm was read by using a UV detector, and the retention time was approximately 5 min. The adsorption capacity (q_e_, mg/g) of erdite was calculated by using the following equation:

13 $${q}_{e}=\frac{({C}_{0}-{C}_{e})\times V}{m},$$

where *C*_0_ and *C*_*e*_ are the initial and equilibrium concentrations of TC (mg/L), respectively, *V* is the solution volume (L), and *m* is the particle dosage (g).

### Characterisation of the erdite nanorods

The morphology of the erdite particles was determined by field-emission scanning electron microscopy (SEM, FEI Co., USA). The X-ray diffraction (XRD) patterns of the erdite particles were analysed by a diffractometer (RAPID-S, Rigaku, Japan) with Cu–Kα radiation in the 2θ range of 10°–40°. X-ray photoelectron spectroscopy (XPS) spectra were obtained using an X-ray photoelectron spectrometer (VG-ADES, England) operated at 150 W with monochromatised Al–Kα X-rays (hν = 1486.6 eV). The base pressure in the analytical chamber was ≈10^−9^ mbar. Narrow-region electron spectra were acquired with an analyser pass energy of 20 eV. Binding energies were calibrated against that of the C1s peak (284.6 eV). The fitting curves were obtained using Thermo Avantage software (version 5.976, Thermo Scientific, USA) with a Shirley background and a Gaussian–Lorentzian peak model.

## Conclusion

Erdite nanorods were synthesised via a facile hydrothermal method using MnO_2_ as an auxiliary reactant. Addition of MnO_2_ considerably promoted the formation of erdite nanorods through the efficient generation of NaOH from the disintegration of Na_2_S. The generated erdite nanorods exhibited a TC adsorption capacity higher than those of previously reported adsorbents. The formation of stable –NH_3_^+^ groups between the hydroxyl group of Fe oxyhydroxide obtained from erdite hydrolysis and the –NH_2_ group in the TC side chain is the major TC adsorption mechanism of erdite.
